# Compensatory muscle activation and spinal curve changes in response to fatigue among adolescent male athletes

**DOI:** 10.1186/s13102-023-00668-6

**Published:** 2023-04-13

**Authors:** Anna Gál-Pottyondy, Bálint Petró, Mária Takács, János Négyesi, Ryoichi Nagatomi, Rita M Kiss

**Affiliations:** 1 Doctoral School of Sport Sciences, Hungarian University of Sports Science, Budapest, Hungary; 2 Faculty of Mechanical Engineering, Department of Mechatronics, Optics and Engineering Informatics, Budapest University of Technology and Economics, Budapest, Hungary; 3 MÁV Hospital, Szolnok, Hungary; 4 Department of Kinesiology, Hungarian University of Sports Science, Budapest, Hungary; 5 Fit4Race Kft, Budapest, Hungary; 6 Department of Medicine and Science in Sports and Exercise, Tohoku University Graduate School of Medicine, Sendai, Japan

**Keywords:** Basketball, Electromyography, Optical tracking, Prone plank test, Spine

## Abstract

**Background:**

The prone plank test has been often used to assess the strength and endurance of trunk muscles. We aimed to develop a new measurement protocol to objectively monitor the changes in spinal curves and muscle activity simultaneously.

**Methods:**

Eleven adolescent male basketball athletes (13–17 years) performed a one-minute plank test. Spinal curvatures (thoracic kyphosis (TK) and lumbar lordosis (LL)) were determined at each time point by optical tracking of markers placed on the spinous processes of 10 vertebrae. Eleven muscles were measured by surface electromyography to determine muscle fatigue via changes in median frequency.

**Results:**

TK significantly increased (*p* = 0.003) from the first to the last 10 s of the plank test; changes in LL were mixed within the group. Only the rectus abdominis showed consistent and significant fatigue (*p* < 0.001). The increased spinal curves significantly correlated with the fatigue of biceps femoris (TK: *r* = -0.75, *p* = 0.012; LL: *r* = -0.71, *p* = 0.019) indicating a compensatory muscle activation and spinal curve changes in response to fatigue.

**Conclusion:**

Our protocol may support future researches that aim to objectively evaluate the prone plank test and which posture-related muscles need strengthening for the individual.

## Background

The optimal activity of several trunk muscles is required to adjust the posture and movement of the body even during different sports activities [1]. Although trunk function is only moderately related to strength and performance [2], it has the potential to predict the development of lower extremity overuse injuries [3]. It is therefore essential to assess trunk function as a part of an athlete’s fitness level; however, appropriate methods for assessing trunk function are not consistent in the literature [4–7]. Most of these tests, including the prone plank (or prone bridge) test [7–9], measure the holding time of a specific posture that has been often used to assess the strength and endurance of trunk muscles. The prone plank test is easy to repeat and requires no tools. However, evaluation is usually done only visually, in a subjective manner by a physiotherapist or other specialist. For example, the test personnel stops the measurement when the hip is perceived to move up or down five centimeters [10], or there are repeated, large deviations from the correct position [11]. Despite the test’s simplicity, results have shown a significant correlation with several athletic performance tests [10]. Adolescent children are going through a critical developmental period characterized by rapid biological changes that also affect core stability. However, considering that both postural control [12] and sagittal spinal-pelvic inclination [13] are superior in early adolescent girls than in boys, probably due to earlier maturation of muscle coordination [14], it seems that within a study only one gender should be focused on.

Surface electromyography (EMG) is often used to assess information about the neural activation of the muscles [15]. Previous studies aimed to determine the activation patterns of the corresponding muscles during a prone plank test via EMG [16–18]. For example, van den Tillaar & Saeterbakken found similar rectus abdominis and obliquus externus abdominis muscle activation during a plank test until failure and 6RM back squats [18], supporting the idea that the prone plank test is an effective method to measure strength and endurance of trunk muscles. Although EMG data can serve as an objective tool for determining fatigue effects on muscle activation during the plank test, measuring the changes in kinematics is also essential to test trunk function [19].

Researchers often apply reflective markers on the skin to determine the position and shape of the spine in natural or specific settings for static or continuous movement [20–23]. Briefly, the participant has reflective markers attached to the skin located at anatomical landmarks [24], i.e., over the spinous processes of different vertebrae, and the positions of the markers are determined by a motion capture (MoCap) system. Although skin marker measurements suffer from uncertainty, they can assess the changes in the lumbar and thoracic curvature angles reliably [23, 25]. A method to determine sagittal spinal curvatures based on skin markers has also been validated recently for static positions [26, 27], considering thoracic kyphosis (TK) and lumbar lordosis (LL). Since MoCap systems allow continuous recordings, the curvature values can be obtained for each timeframe.

Currently, there is no consensus on how to evaluate the prone plank test. We hypothesize that tracking the spinal curvature changes would offer an objective method to determine the outcome of the prone plank test while obtaining muscle fatigue data would help identify posture-related muscles that need further conditioning. Our goal is to develop and test an experimental protocol that has the potential to objectively evaluate the changes in spinal curves and muscle activation in response to fatigue during the plank test. To determine the degree of muscle fatigue, we calculated the decrease in median frequency (MDF) [28, 29].

## Materials and methods

### Participants

Eleven adolescent male athletes (age = 14.6 ± 1.4, range 13–17 years; height = 181 ± 13.8, range 153–199 cm; mass = 63.1 ± 12.8, range 37–81 kg; basketball-specific training load: 12.8 ± 1.7 h/week; strength training load: 2.1 ± 0.2 h/week; basketball career: 6.9 ± 1.8 years; playing time: 20.5 ± 8.2 min/game) of the Szolnok Basketball Academy participated in this study. Participants were included if they were (1) certified basketball players, (2) licensed by a sports medicine doctor, (3) < 18 years old, (4) male, (5) had at least 9 h of training per week, and (6) healthy. Exclusion criteria within this population were (1) injury within 6 months, (2) pain, and (3) orthopedic or neurological disorder. After giving both verbal and written explanations of the experimental protocol to the participants, their parent(s), and trainer(s), all participants and their parents/guardians signed informed consent prior to the start of the experiment that was executed in accordance with the Declaration of Helsinki. The study was carried out in accordance with the recommendations of the University Ethical Committee. The examinations were authorized by the Research Ethics Committee of the Hungarian University of Sports Science, Hungary (license number TE-KEB/No6/2019). The parent(s) or the trainer(s) were also present during the experiment, given that electrodes were placed on the adolescents’ skin areas that are of a sensitive nature.

### Experimental procedures

**Fig. 1:**
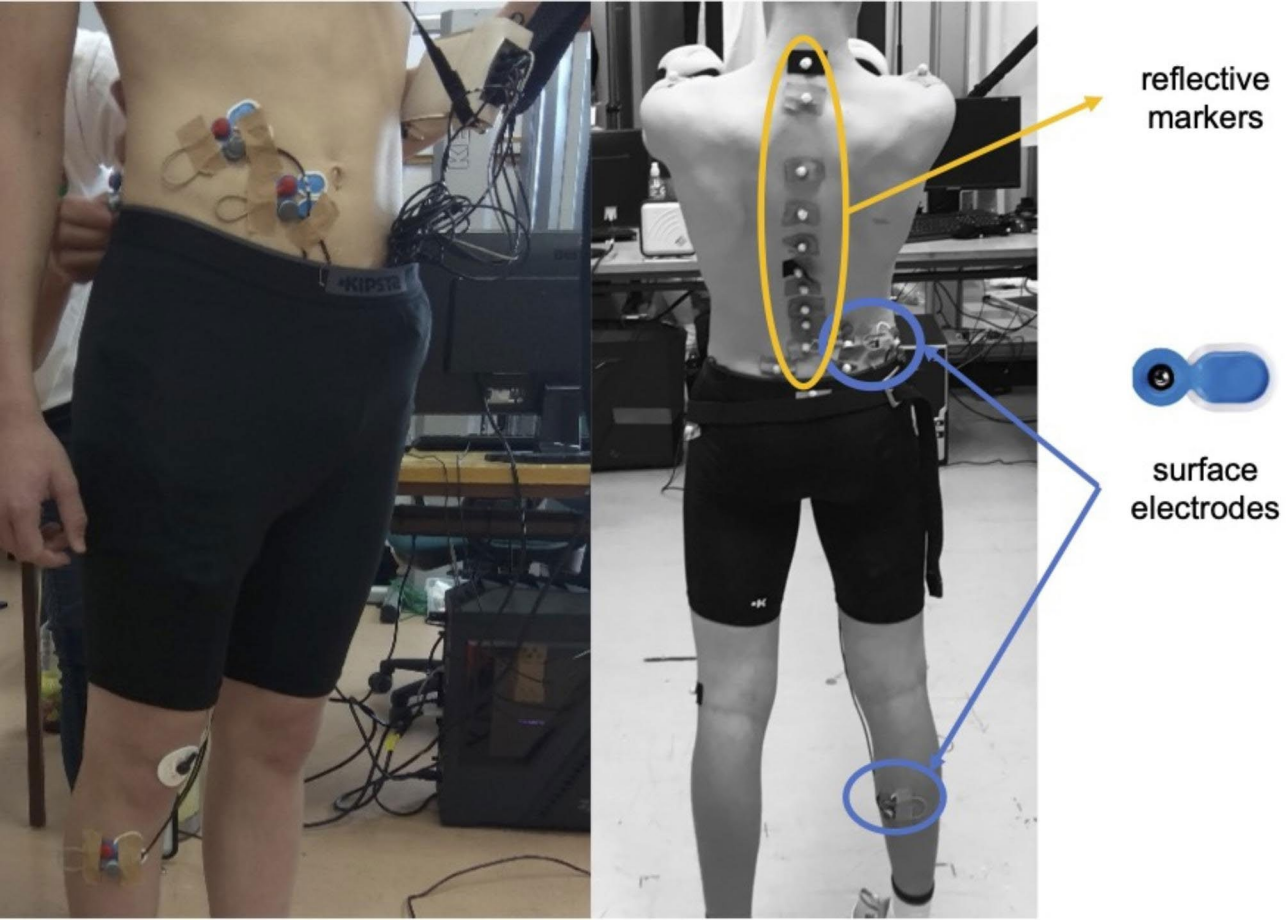
The placement of reflective markers and surface electrodes

**Fig. 2:**
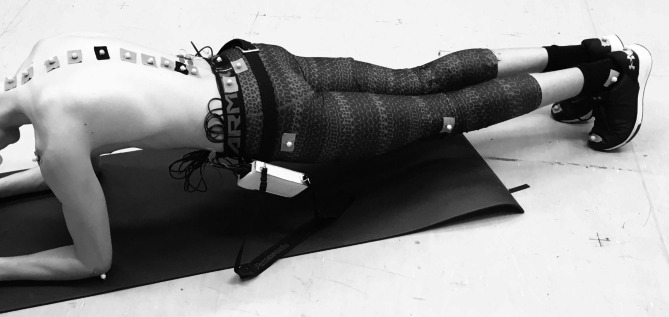
The prone plank position. Reflective markers were placed on spinous processes of 10 vertebrae and the sacrum

After applying the EMG electrodes and reflective markers to the participant’s body (Fig. 1), he was asked to hold the prone plank position (Fig. 2) for one minute. The data acquisition started when the athlete held the correct position. The instruction before the test was: (1) begin the plank position, (2) face down with your forearms and toes on the floor, your elbows are directly under your shoulders and your forearms are facing forward, (3) your head is in line with the spine and you are looking at the floor, (4) your body is straight: the nape, the part of your back between your shoulders, the sacrum, the knees, and the ankles fit in one line, (5) hold this position as still as you can for one minute. No other instruction was given during data acquisition, even if the posture was not correct anymore. The MoCap system was connected and synchronized with the EMG acquisition system, allowing us to simultaneously monitor the changes in spinal curves and muscle activity.

#### Measuring the angles of spine curves

Retroreflective markers were placed on the participant to measure the shape of the spine and the body’s position. The spatial position of the markers was monitored with an 18-camera OptiTrack© Motive (NaturalPoint Inc., Oregon, USA) MoCap system at a sampling rate of 100 Hz. The markers were attached over the skin on the spinous processes of the following ten vertebrae: C6, T1, T4, T6, T8, T10, T12, L2, L4, and L5 (Fig. 2). For better traceability, additional markers were placed on the sacrum, near the right and left ears, on the tip of the shoulders, on the lateral epicondyles of the elbows and wrists, on the greater trochanters, and on the lateral sides of the knee and ankle. During data processing, the measured marker positions were smoothed using a low-pass filter with a cutoff frequency of 6 Hz. To determine the shape of the spine, we used a recently validated method [26]: a spatial spline curve was fitted to the ten markers by minimizing the value of square error. Angles of TK and LL were calculated as the angles between tangents of the spline fit at T1 and T12, and T12 and L5, respectively [27].

#### Electromyography

A 16-channel NORAXON (Noraxon Inc., Scottsdale, AZ, USA) EMG system with telemetry (Fig. 1) connected to a 16-channel amplifier was used to assess EMG activity of the 11 selected muscles, including erector spinae longissimus, erector spinae iliocostalis, obliquus externus abdominis, rectus abdominis, gluteus maximus, gluteus medius, rectus femoris, adductor longus, biceps femoris, tibialis anterior, gastrocnemius medialis. EMG was sampled at 2 kHz and the signal conditioning circuit included analog filtering with a bandwidth of 0–1150 Hz. This filter is an analog filter in the signal conditioning circuit.

Bipolar silver-silver chloride (Ag-AgCl) surface electrodes (Ambu, Ballerup, Denmark) were positioned at an interelectrode distance of 2 cm based on the recommendations published by SENIAM (Surface EMG for Non-Invasive Assessment of Muscles) [30]. During the positioning of the electrodes, we considered (1) the anatomy of the muscle by sight and palpation, (2) the elecrodes’ signal receiving area, and (3) the recommendation of Seniam. After placing the electrodes, we checked the signals and, if deemed necessary, corrected the location of the electrodes.﻿ A ground electrode was placed over the patella. Before the electrodes were attached, the attachment sites were shaved and then cleaned with an alcohol swab. The electrodes and their cables were attached to the skin using clinical tape (Leukoplast Universal Tape, BSN Medical GmbH, Hamburg, Germany). A high-pass FIR filter of 20 Hz cutoff frequency (tap size 120) and a low-pass FIR filter of 500 Hz cutoff frequency (tap size 120) were applied in a zero-phase manner to filter interference signals and extract the relevant signals. Figure 4 shows an example of the raw signals.

**Fig. 3:**
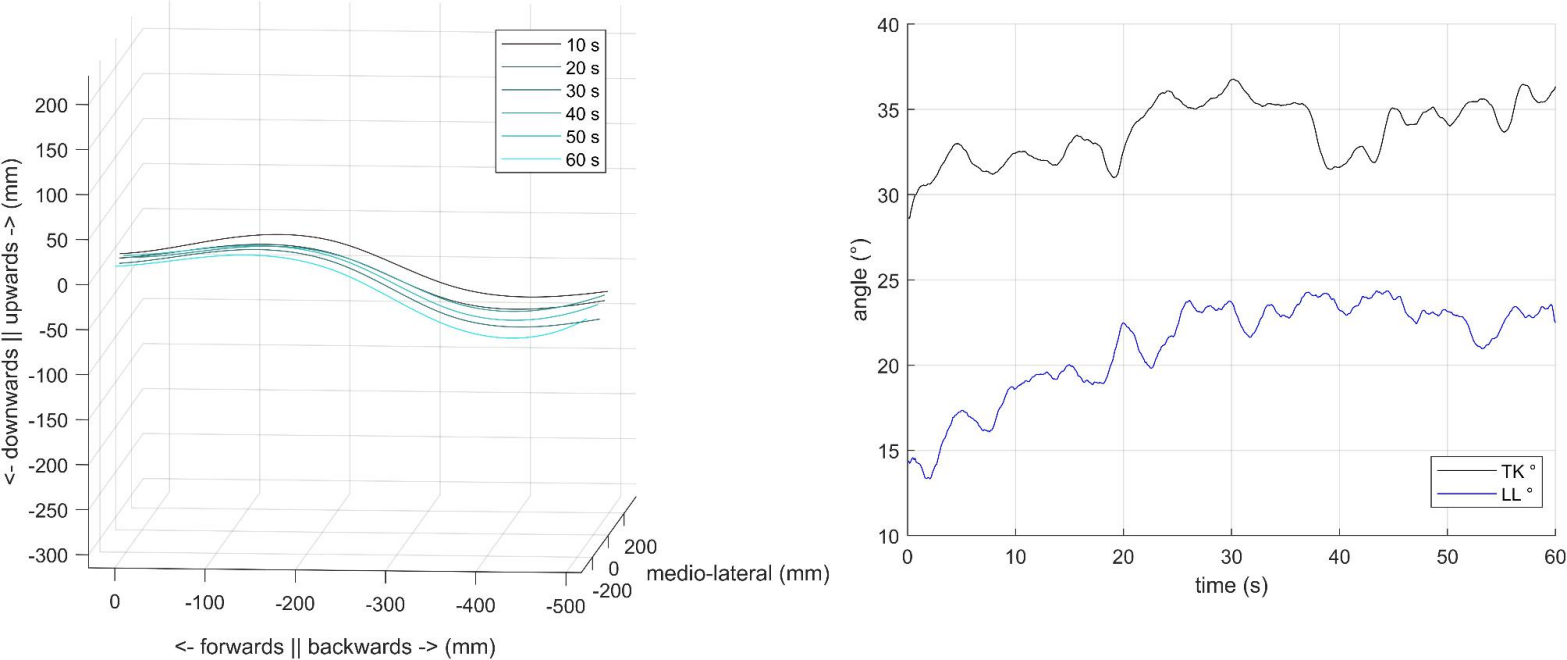
Changes in spinal curves through time during the prone plank test from a representative participant. *Left*: The shape of the spine at each subsequent 10 s of the test. *Right*: Changes in thoracic kyphosis (TK, black line) and lumbar lordosis (LL, blue line) angles through time

A decrease in the power spectrum MDF indicates fatigue [28, 31, 32]. Applying the short-time Fourier transform requires the signal window (epoch) to be stationary. According to [31], for an isometric constant force contraction, EMG signals can be considered wide-sense stationary as long as they exclude fatigue (p137) and recommends the use of non-overlapping epochs of 0.25 to 1 s (p146). Farina and Merletti [33] found that epoch durations between 250 and 500 ms produce the most suitable stationarity properties. Thus, the 60 s recordings were divided into 500 ms epochs without overlaps, and the stationarity of these epochs was checked by the Kwiatkowski, Phillips, Schmidt, and Shin (KPSS) test via the Matlab function ‘kpsstest’. The MDF value between the [50, 500] Hz interval was obtained for these epochs using the ‘medfreq’ function of Matlab. In order to compare different muscles and to be able to interpret the fatigue as the slope of the fitted line, the MDF values were 0-100 normalized to the maximum frequency values of each muscle for each recording. A linear regression curve was fitted to the normalized MDF values. The slope of the regression line represented the average change in the MDF. A negative slope was considered to indicate muscle fatigue, with a larger negative slope corresponding to a greater degree of muscle fatigue.

### Statistical analyses

The analyses were performed using SPSS Statistics Package (version 22.0, SPSS Inc., Chicago, IL). Normal distribution of the data was checked using the Shapiro-Wilk test and visual inspection of the histograms. Although most of the data were normally distributed, non-parametric statistical analyses were used due to the relatively small sample size. Thus, Wilcoxon sign-rank tests were performed to statistically investigate the changes in TK and LL spinal curves in response to fatigue during the prone plank test and whether changes in MDF (the slope of the regression curve) differ from zero. Additionally, the *r* Wilcoxon effect size was also computed as appropriate. In order to determine if changes in spinal curves were associated with changes in the activity of the 11 selected muscles, Spearman’s correlations were computed between the changes in TK, LL, and fatigue values. Note that the correlation value between muscle fatigue and TK or LL is negative if an increase in spinal curve angles coincides with greater fatigue. Statistical significance was set at *p* < 0.05.

## Results

Figure 3 shows the changes in spinal curves and the evolution of curvature values through time during the prone plank test from a representative participant. For the group, Fig. 5 shows the changes in TK and LL values. TK significantly increased from the first to the last 10 s of the plank test (*T* = 0; *Z* = -2.934; *p* = 0.003; *r [Wilcoxon effect size]* = 0.88), while LL angles either slightly reduced or greatly increased, which prevented us from detecting significant changes (*T* = 24; *Z* = -0.800; *p* = 0.424; *r [Wilcoxon effect size]* = 0.24) (Fig. 5, b). Although changes in TK and LL failed to show significant correlation (*r*_*S*_ = 0.573; *p* = 0.066), the scatterplot (Fig. 5b) clearly illustrates that they were strongly associated with each other besides the two outliers. However, there were no apparent connections between the initial values and the changes in response to the test (Fig. 5a).

**Fig. 4:**
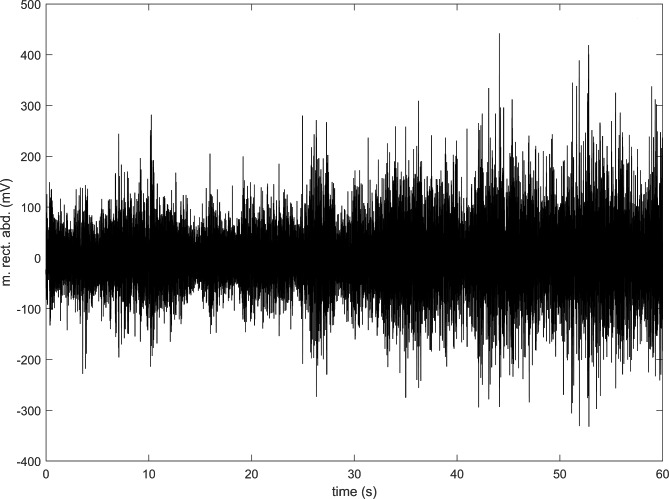
Raw EMG signal of musculus rectus abdominis during 1-minute plank test

**Fig. 5:**
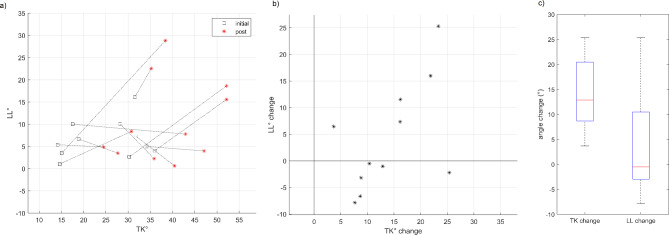
**(a)** Scatterplot representing the changes in thoracic kyphosis (TK) and lumbar lordosis (LL) angles for each participant with initial and post-exercise values and **(b)** scatterplot of the changes in the angles. **(c)** Boxplot of changes in thoracic kyphosis (TK) and lumbar lordosis (LL) angles. The boxplots show the median, the upper and lower quartiles, and the min and max values of the age groups, * *p* < 0.05

**Fig. 6:**
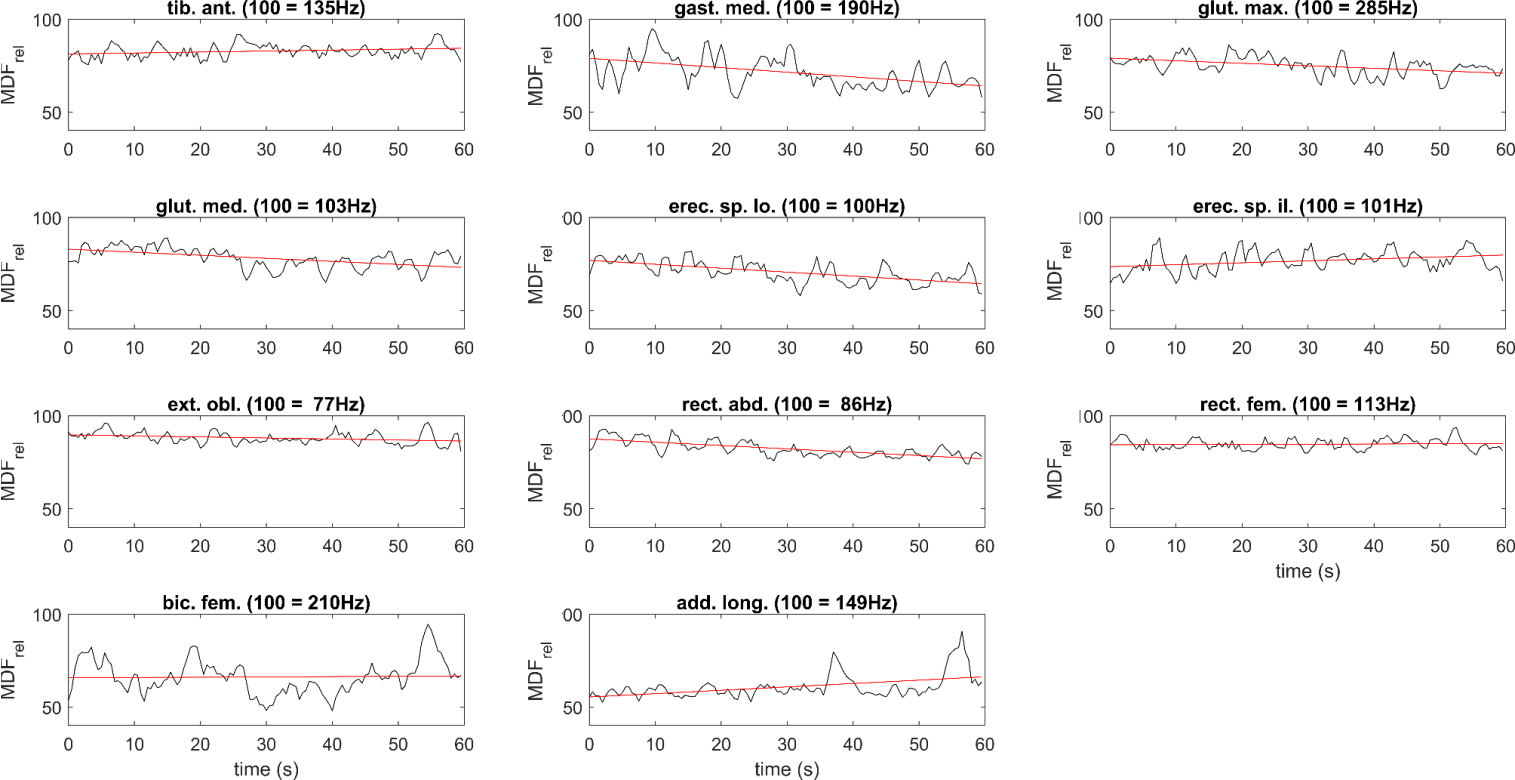
The average change in the median frequency (MDF) of the 11 selected muscles’ EMG from a representative participant. MDF traces were smoothed by a 3-size moving average window to increase visual clarity. A decreasing slope of the regression line (red) was considered muscle fatigue, where a greater negative slope corresponded to a greater degree of muscle fatigue

The MDF of most of the measured muscles’ EMG activity showed various behaviors across the group (see example on Fig. 6); fluctuation around a constant value, steady decrease, and steady increase could all be observed during the prone plank test. Some muscles also exhibited a widely fluctuating MDF. In the participant group, these were typically the gluteus maximus, gluteus medius, gastrocnemius medialis, and biceps femoris. As an example, note the gluteus maximus, the gastrocnemius medialis, and the biceps femoris MDF in Fig. 6. The stationarity test indicated that 0.38% of epochs were non-stationary on average; the muscle with the highest level of non-stationarity was the erector spinae longissimus (2.67% of epochs on average).

**Fig. 7:**
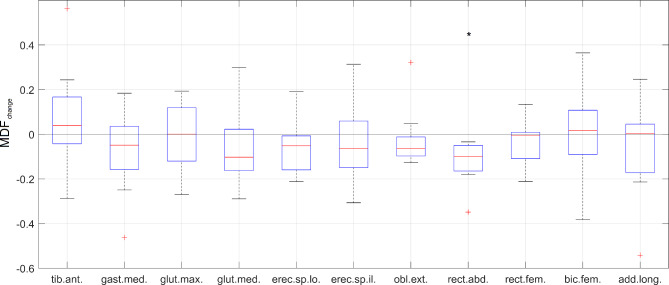
The degree of muscle fatigue in the selected 11 muscles in response to the 60-sec prone plank test. The boxplots show the median, the upper and lower quartiles, and the min and max values of MDF changes across the group; * marks significantly non-zero median values at *p* < 0.05

A significant level of fatigue in response to the 60 s prone plank test (Table 1) was observed only in the case of the rectus abdominis (*p* < 0.001); the slope of fatigue was negative for all participants (Fig. 7). In the case of obliquus externus abdominis, nine of the eleven participants showed a negative MDF slope; however, the two positive values prevented us from finding a statistically significant non-zero fatigue (*p* = 0.102). The MDF slope values showed a wider range for the other measured muscles, encompassing both positive and negative values (Fig. 7).

**Table 1:** The level of fatigue (p-values of the Wilcoxon sign-rank tests) in the measured muscles, and their correlation with the changes in spinal curves

Muscle	Significance of fatigue (*p-values*)	Correlations (*r*_*S*_)	
		*TK*	*LL*
*erec. sp. lo.*	0.123	0.02	-0.35
*erec. sp. il.*	0.365	-0.21	0.39
*obl. ext. abd.*	0.102	0.01	0.49
*rect. abd.*	< 0.001	0.34	0.02
*glut. max.*	0.898	-0.11	-0.25
*glut. med.*	0.175	0.19	0.12
*rect. fem.*	0.365	0.23	0.32
*add. lon.*	0.700	0.03	0.15
*bic. fem.*	0.898	-0.74*	-0.71*
*tib. ant.*	0.413	-0.48	-0.42
*gast. med.*	0.240	0.19	0.12

*erec. sp. lo.*, erector spinae longissimus; *erec. sp. il.*, erector spinae iliocostalis; *obl. ext. abd.*, obliquus externus abdominis; *rect. abd.*, rectus abdominis; *glut. max.*, gluteus maximus; *glut. med.*, gluteus medius; *rect. fem.*, rectus femoris; *add. lon.*, adductor longus; *bic. fem.*, biceps femoris; *tib. ant.*, tibialis anterior; *gast. med.*, gastrocnemius medialis; *r*_*S*_, Spearman’s correlation coefficient; *TK*, thoracic kyphosis; *LL*, lumbar lordosis; *p < 0.05

Few significant correlations were found between muscle fatigue and spinal curve changes (Table 1). The level of fatigue for the rectus abdominis did not correlate with changes in either of the spinal curves (TK: *r* = 0.34, *p* = 0.30; LL: *r* = 0.01, *p* = 0.96), despite being the only muscle showing significant fatigue across the group. The increase in spinal curves significantly correlated with the fatigue of biceps femoris (TK: *r* = -0.75, *p* = 0.012; LL: *r* = -0.71, *p* = 0.019) (Table 1), indicating a compensatory muscle activation and spinal curve changes in response to fatigue.

## Discussion

The present study aimed to develop and test an experimental protocol that has the potential to objectively evaluate the changes in sagittal spinal and muscle activation in response to fatigue during the plank test using a MoCap system and EMG, respectively. Our results indicated that our recently validated method [26, 27] could be effectively used for tracking spinal curvature changes. A significant increase in TK angles from the first to the last 10 s of the plank test was found. We also determined the degree of muscle fatigue by calculating the MDF of the measured muscles’ EMG activity. Significant fatigue in response to the 60 s prone plank test was observed only in the case of the rectus abdominis; the fatigue of the other muscles varied considerably. Finally, the data indicated compensatory muscle activation and spinal curve changes in response to fatigue.

While TK significantly increased from the first to the last 10 s of the plank test in each participant during the 60 s prone plank test, changes in LL angles showed inconsistent results. Some of the participants straightened the lumbar area of the spine which corresponded to slightly reduced LL angle values, while others greatly increased LL angles by tilting the pelvis, suggesting that participants used different strategies in response to the demands of the test. This is in line with a previous study [11] that observed several manifestations of fatigue during a plank test, including shaking, back pain, and abdominal and thigh fatigue. The observed great increase in LL may be an indicator of failing to complete the prone plank test in case of fixed time duration or may mark the end time of successful execution. Consequently, the application of a MoCap system during the prone plank test can be a useful tool to objectively determine the outcome of the postural muscle test based on spinal curvatures and the motor control strategies in response to fatigue in order to detect trunk function.

In the present study, MDF for most muscles showed various behaviors (increase, decrease, or constant with slight fluctuations) across the group. In addition, gluteus maximus, gluteus medius, gastrocnemius medialis, and biceps femoris tended to show largely fluctuating activities during the test. The statistical analyses on the MDF slopes indicated a significant level of fatigue for the rectus abdominis muscle (*p* < 0.001) in response to the 60 s prone plank test, while the same for the externus obliquus abdominis was not significant (*p* = 0.102). This is not in line with the study from Czaprowski et al. [34], which found that the obliquus externus abdominis muscle had the highest EMG activity (42.3 ± 19.5% of MVC) during a prone plank test. Nevertheless, Escamilla et al. [35] showed an almost identical muscle activation for the rectus abdominis (40 ± 10% of MVC) and obliquus externus abdominis (40 ± 20% of MVC) during a plank test, suggesting that both muscles play a significant role in maintaining the plank position and are essential for trunk function. Further studies should determine whether their activation level was associated with sports performance.

Although only the changes in the MDF of rectus femoris correlated with the changes in spinal curves, our results support the idea that the proposed experimental setup has the potential to objectively determine the fatigue-induced changes in the kinematics and muscle activation during a prone plank test using a MoCap system and EMG, respectively. For example, a substantial change in the TK curve at ~ 40 s in Fig. 3 can be explained by the decreased activation of gluteus maximus, biceps femoris, and erector spinae iliocostalis (Fig. 6). It is possible that during a 60-second-long exercise, the activation of the involved muscles within a muscle group (e.g., abdominal muscles, hip flexors, hip adductors, knee flexors, etc.) changes through time to reduce fatigue. Therefore, grouping the muscles by function and analyzing their activity together are recommended to clearly detect whether changes in muscle activation in response to fatigue are associated with spinal curve changes.

Limitations arise from the small sample size and the use of surface electrodes which prevented the detection of the activity of deeper posture-related muscles. Future studies need to recruit more participants of different ages and sports. Another limitation was the test’s fixed duration, which may not have induced fatigue in some individuals. This can be avoided by testing until failure. Our experimental setup could also be applied in other trunk function tests, e.g., the Matthias test, and can involve other joints. Moreover, grouping the muscles by function and analyzing their activity altogether may allow us to detect the participants’ voluntary and involuntary motor control strategies. Future studies may also consider determining the role of shoulder and arm muscles when increased kyphosis due to fatigue is present during the prone plank test.

## Conclusions

In conclusion, we found a significant increase in thoracic kyphosis angles from the first to the last 10 s of the plank test. Lumbar lordosis angles slightly decreased for some participants while markedly increased for others. Consistent and significant fatigue was observed across the group only in the case of the rectus abdominis, but the level of fatigue did not correlate with spinal curve changes. However, biceps femoris fatigue significantly correlated with the changes in kyphosis and lordosis angles to a large degree. Moreover, the data indicated compensatory muscle activation and spinal curve changes in response to fatigue. These preliminary results suggest that the proposed method has the potential to objectively detect the strength of posture-related muscles during the prone plank test. Our experimental protocol may support future researches that aim to objectively detect the strength of posture-related muscles via any other trunk function test at any age group and sport and identify muscles that need further conditioning on an individual level.

## Data Availability

The datasets generated during and analyzed during the current study are not publicly available due to the large amount of EMG and motion capture data with associated excel files but are available from the corresponding author on reasonable request.

## Abbreviations

EMG: Electromyography
LL: lumbar lordosis
MoCap: motion capture
TK: thoracic kyphosis
SENIAM: Surface EMG for Non-Invasive Assessment of Muscles
